# The Effect of Feeding Laying Hens with Nonindustrial Hemp Seed on the Fatty Acid Profile, Cholesterol Level, and Tocopherol Composition of Egg Yolk

**DOI:** 10.1155/2023/1360276

**Published:** 2023-05-27

**Authors:** Yassine Taaifi, Kamal Belhaj, Farid Mansouri, Youssef Rbah, Reda Melhaoui, Nadia Houmy, Abdesammad Ben moumen, Embarek Azeroual, Mohamed Addi, Ahmed Elamrani, Hana Serghini-Caid

**Affiliations:** ^1^Laboratory for Agricultural Production Improvement, Biotechnology and Environment, Faculty of Sciences, Mohammed I University, BP-717, 60000 Oujda, Morocco; ^2^Laboratory of Sustainable Agriculture Management, Higher School of Technology Sidi Bennour, University Chouaib Doukkali, Street Jabran Khalil Jabran BP, 299-24000 El Jadida, Morocco; ^3^Higher School of Education and Training, Mohammed I University, BP-410, 60000 Oujda, Morocco; ^4^Royal Institute of Livestock Fouarat, Kenitra, Morocco

## Abstract

The purpose of this study was to evaluate how cannabis-derived nonindustrial hemp seed (HS) inclusion in laying hen diets, as well as treatment duration, affected the fatty acid (FA) profile, cholesterol level, and tocopherol composition of egg yolks. Ninety-six (*n* = 96) Lohmann Brown classic laying hens were randomly assigned to one of the four groups: control (standard diet) and HS-containing diets (10% HS, 20% HS, and 30% HS). The study was conducted for a period of 4 months. The findings demonstrated that the FA profile and the tocopherol composition are strongly impacted by the addition of HS to the diet of laying hens (*p* < 0.05), but the cholesterol content remained unaffected. The increase in the dose of cannabis incorporated into the hen's diet (HS-30% group) led to a significant increase in the amounts of the polyunsaturated fatty acids n-3 and n-6 content of egg yolk. This enrichment was accompanied by a considerable decrease in the n-6/n-3 ratio (*p* < 0.001) from 8.19 to 4.88, on day 84 of the experiment. The total tocopherol content significantly increased (*p* < 0.05) from 281.44 (control) to 327.02 *μ*g/g yolk (HS-30%) on day 84. Finally, in the context of warfare, these seeds might be used as a feed additive for laying hens to produce higher nutritive value eggs with affordable prices.

## 1. Introduction

The fatty acid (FA) content of animal-based foods, such as meat, dairy products, and egg yolks, is a perfect reflection of the lipid profile and the constitution of nutrition. Due to the biohydrogenation phenomena, this association is more frequently found in monogastric [1] than polygastric ruminants [2]. Because polyunsaturated fatty acids (PUFAs) are vital for humans, there is a growing awareness of their health advantages [3]. Furthermore, the n-6/n-3 polyunsaturated fatty acid (PUFA) ratio remains an important criterion for assessing fat quality and should be less than the value of 4 [4]. Moreover, nutritional survey shows that PUFA n-6 predominates over PUFA n-3 by a factor of 10 to 30, which indicates a PUFA n-3 deficiency. These latter nutrients are crucial for human health and important in preventing behavioral issues, diabetes, some cancers, inflammations, and cardiovascular disease (CVD) [5].

Furthermore, international health experts advise consuming enough PUFAs, namely, eicosapentaenoic acid (EPA) and docosahexaenoic acid (DHA), mostly found in fishery products. Nevertheless, this recommendation is not applicable in countries with low-income workforce (such as Morocco), where fish consumption is low due to its limited availability and high price. However, eggs are consumed more frequently in almost all countries around the world due to their availability to the consumers as well as their low cost. Therefore, enriching eggs with PUFA n-3 is a sustainable option for a healthy and well-balanced diet, as well as a way to reduce the intensive exploitation of fishery resources [6].

For a long time, egg consumption has been one of the simplest and cheapest ways to provide populations with the essential nutrients for a healthy and balanced diet (protein, fat, vitamins, and minerals). Eggs have been prevented from consumption by dieticians for a long time due to their high cholesterol and saturated fatty acid content [1]. Alexander et al. [7] reported in an interesting review that there are substantial correlations between cholesterol intake and heart disease. However, recent studies have revealed that there is minimal up to no connection between dietary cholesterol and cardiovascular risk indicators due to the interaction of several other factors of risk such as a sedentary lifestyle, smoking, obesity, and other physiological dysfunctions [7]. According to several studies, animal nutrition has a significant impact on both biochemical constitution and its nutritional value [8, 9].

Further studies found that the incorporation of n-3- and tocopherol-rich nutrients in laying hens' diet produced eggs with higher levels of these constituents and thus higher nutritional value [10]. Furthermore, incorporating flaxseed and fish oil in farm livestock feed items is a frequent method for enhancing meat with n-3 PUFA and eggs [11–13]. Nowadays, the development of innovative and alternative methods based on new natural plant resources would be of considerable interest.

*Cannabis sativa* seeds and derivatives have a long history of usage in ethnomedicine [14]. Recent research has shown interest in using cannabis seeds as a supplement of livestock feed, particularly as a source of PUFA n-3 [10, 15, 16]. These studies focused on supplementing the animal's diet with industrial hemp seed, seed meal, or flour, which revealed an increase in n-3 content while reducing the n-6/n-3 ratio. In addition, hemp seeds increased hen laying performance, such as the egg laying rate, as well as egg quality, namely, the average egg weight, eggshell strength, and the biochemical composition. Furthermore, Kasula et al. [17] showed that laying hens fed hemp seedcake have a high polyunsaturated fatty acid content as well as no tetrahydrocannabinol or cannabinoid residues in eggs, internal organs, or body tissue indicating that there are no transfer cannabinoid residues in hens' fats. According to a survey conducted in Morocco [18], 80% of the participants thought that the availability of eggs fortified with n-3 would be interesting, and 74% stated that they would be willing to pay more for them than ordinary eggs.

Therefore, the objective of this research was to investigate how the incorporation of the Moroccan nonindustrial cannabis seed affects the composition of tocopherols, cholesterol, and the fatty acid profile of yolk eggs. For this purpose, experiments were conducted using different ratios of nonindustrial cannabis seeds in a laying hen diet over different periods. The findings of this study will support the industry in reducing imports of more expensive oilseeds, such as soya, sunflower, and maize, and improving feed formulation to produce eggs that are n-enriched utilizing Moroccan nonindustrial cannabis seeds.

## 2. Material and Methods

### 2.1. Experimental Animals

The animal experiment was conducted in accordance with the European regulations CO 74/99 regarding stocking density, lighting, ventilation, and vaccination, at the Royal Institute of Livestock in Kenitra, Morocco. The trials on laying hens were carried out at the Royal Institute specialized in breeding of Kenitra, Morocco, in conformity with the European code number 74/99. The layers used in this study were randomly distributed in four groups of the Lohmann Brown classic (LBC) strain repeated six times (4 × 4 × 6 = 96), including a control group. During the rearing period, no new husbandry practices were introduced. The hens were raised in a semiautomatic coop in cages with the following characteristics: 61 cm long, 57 cm wide, and 50 cm high. The birds were housed with 4 hens per cage equipped with feeders and nipple drinkers, the building being equipped with automatic dejection mats. The environmental parameters were managed according to the guidelines of the exploited strain, with an average temperature between 18 and 20°C, humidity between 55 and 60%, and light duration of 16 hours/day, from 6:00 to 22:00. The laying hens come from a modern certified farm, at the age of 22 weeks.

### 2.2. Diets and Experimental Approach

To standardize the energy level of 3000 kcal/kg, ingredients were added to the hen's foods to generate diets with similar levels of nutrient composition. The formulation of the diet was developed in consultation with BENWAY, a company specializing in poultry feed. The feed has been formulated in collaboration with BENWAY, a company specialized in the feed manufacture for laying hens. Maize-/soya bean-based diets were utilized by the inclusion of different hemp seed levels of the local ecotype “Beldiya”: 0.0% (HS-0) control, 10% (HS-10), 20% (HS-20), and 30% (HS-30). The inclusion of hemp seeds of the local ecotype “Beldiya” on a feed composed of corn/soybean was on four levels: 0.0% (HS-0) control, 10% (HS-10), 20% (HS-20), and 30% (HS-30).  Table 1 shows the HS compositions utilized in this investigation. The chemical composition of the cannabis-based diet used in this study was described in our previous article (Table 2) [19]. For two weeks, the hens were given time to adapt to their new environment system (cage and feeding method). In the first week, hens were given a commercial layer meal, and in the second week, the experimental diets were gradually introduced from 25%, 50%, and 75% to finally 100%. In the first week, the birds were fed a standard layer meal, and in the next week, the experimental regimes were gradually introduced at 25%, 50%, 75%, and finally 100%.

The birds were fed three times a day while given unlimited access to water. The study was conducted for a period of 4 months (from January 30th to the 31^st^ of May 2021). Eggs were randomly sampled during the last three days of each period (28 days/period). 18 eggs per group (3 eggs × 6 replicates) were collected for a period of 3 consecutive days at 28, 56, and 84 days of treatment corresponding to 28, 32, and 36 weeks of age. The eggs were analyzed for lipids, fatty acids, cholesterol, and tocopherols.

### 2.3. Chemical Analysis

#### 2.3.1. Diet and Egg Yolk Fatty Acid Extraction

Three eggs were randomly selected from each treatment with 6 replicates. Yolk and white were carefully separated to avoid mixing. The 3 yolks were pooled and homogenized in a 50 mL tube and stored at -20°C until analysis time. The four tested diets, on the other hand, were powdered and kept at -20°C for chemical composition analysis. Lipid extraction from all the samples was performed according to the method described by Bligh and Dyer [20] using a mixture of chloroform/methanol/water solvent (2/1/1; *v*/*v*/*v*). The solvents were evaporated using a Hahnvapor Rotary Evaporator HS-2005S-N (AM-486). The extracts were tested for fatty acids, cholesterol, and tocopherols.

#### 2.3.2. Fatty Acid Profile

The fatty acids were converted into fatty acid methyl esters (FAMEs) before being analyzed using our previously published method [21]. The composition of FAME was evaluated using gas chromatography (GC Agilent 6890, Agilent Technologies) coupled with a flame ionization detector (FID). FAMEs (injection of 1 *μ*L of the sample in splitless mode) were separated on a BPX70 capillary column with the following characteristics (60 m length, 0.32 mm internal diameter, and 0.25 *μ*m film thickness; SGE Europe). Helium was used as carrier gas at a flow rate of 1 mL min^−1^. The oven's temperature was set at 50°C and afterwards increased to 170°C at a rate of 30°C per minute and then increased by 4°C per minute to 220°C. The temperature was then held for 10 minutes. After identifying the fatty acids and comparing them to a standard from Sigma-Aldrich containing 37 FAMEs (Supelco, Bellefonte, PA, USA), the results were presented as percentages. Other sums of FA are determined as odd-chain fatty acids (OFA) and have beneficial effects on human health [22–25]. The desirable fatty acids (DFA) are also calculated by the following formula: DFA = (C18 : 0 + UFA) according to Vlaicu et al. [13] and Belhaj et al. [26]. These latter are considered as hypocholesterolemic FAs by reducing the low-density lipoprotein impacts [22].

#### 2.3.3. Tocopherol Content

Tocopherols were quantified according to the Ben Moumen et al. [27] protocol using an HPLC system (Shimadzu LC-6AD system) coupled with a DAD detector. The separation was carried out on an Uptisphere 120 A NH2 silica column (4.6 × 250 mm, the particle size of 5 *μ*m) using a mobile phase composed of an n-hexane/isopropanol mixture (99/1; *v*/*v*) with a flow rate of 1 mL min^−1^. The identification was carried out using commercial standards for tocopherols (Sigma-Aldrich, St. Louis, USA) at 292, 296, and 298 nm. The tocopherol concentration was calculated from the external calibration curve with commercial tocopherols obtained from Sigma-Aldrich (St. Louis, MO, USA).

#### 2.3.4. Cholesterol Content

The cholesterol content of egg yolk was measured using the method described by Vanderplanck et al. [28]. After saponification with KOH and extraction with diethyl ether, cholesterol was separated from the fat. The mixture was analyzed by gas chromatography using a Hewlett-Packard chromatograph (HP 6890 series GC) equipped with a capillary column (HP 5 MS (5 %-phenylmethylpolysiloxane, 30m × 0:25mm, 0.25 m film thickness), Agilent Technologies, Palo Alto, CA, USA) and a flame ionization detector. The injector was operated in splitless mode. The operating parameters were as follows: carrier gas: helium at 1 mL min^−1^; column temperature: 275°C; injector and detector temperatures: 250 and 300°C, respectively; and injection volume: 1 *μ*L. The cholesterol concentration in egg yolk was calculated and expressed as mg per g of egg yolk.

### 2.4. Statistical Analysis

The statistical analyses were carried out using the Statistical Package for the Social Sciences (IBM SPSS 21). The normal distribution was checked using the Shapiro-Wilk test for quantitative variables. A two-way analysis of variance (ANOVA) was performed for fatty acid profile, tocopherol content, and yolk cholesterol content. The Tukey post hoc test was used for the comparison of means. The difference was considered significant at *p* < 0.05. A principal component analysis (PCA) was performed on the data set to differentiate the results according to the age of the hens and the feed distributed.

## 3. Results and Discussion

### 3.1. Fatty Acid and Cholesterol Composition

The results in Tables 3–6 show the effect of Moroccan nonindustrial hemp seed incorporation in the diet of laying hens on the fatty acid profile of the egg yolk during the experimental period. The results obtained show varying fluctuations concerning the dose and duration of the experiment; however, the main variations concern the increase in ALA content (Tables 3–6). We note a statistically significant difference (*p* < 0.05) in the amount of n-3 in the eggs depending on the diet used throughout the experiment and the amount of cannabis seeds supplied. After 84 days (12 weeks) of the feeding period, which corresponded the end of the experiment, the n-3 content increased as the incorporated dose of cannabis seeds increased (Tables 3 and 5). Thus, the percentage of n-3 escalated from 3.01% in the control (0% HS) to 3.41, 5.45, and 7.05% in the eggs from the hens fed, respectively, with 10%, 20%, and 30% of cannabis seeds (Table 5). The detailed analysis of fatty acids reveals that this increase in n-3 correlates specifically with an increase in ALA, with the level rising from 1.62% (control) to 5.47% (HS-30%). However, there are no significant differences in DHA and EPA levels. This last result contradicts most of the previously published results (Fabro et al. [29]; Mierliță [30]. The long-chain AGPI metabolism in animals allows the conversion of ALA to DHA and EPA, only if the ratio of n-6 to n-3 is not too high; otherwise, the path of converting LA to ARA will be promoted. In contrast and based on our results, the ARA rates no longer exhibit significant and noticeable variations. These results could be explained by a potential oxidative degradation of DHA, EPA, and ARA (long-chain PUFA) during the extraction of the lipid phase from egg yolks and throughout the other phases of the fatty acid analysis of. In fact, no antioxidants were added during the different phases of the lipid analysis, compared to other studies [13]. This deliberate decision to exclude the antioxidant agents was made to mimic the conditions of an egg consumer or an industry who would be using products made from raw eggs. Moreover, other experiments are being conducted in our laboratory to study the effect of the antioxidant's addition on the fatty acid composition (data not shown). Furthermore, other studies on chicken fed cannabis seeds or oil revealed a significant reduction in the expression of gene coding for hepatic desaturases responsible for long-chain PUFA desaturation [31]. The incorporation of cannabis seeds in poultry feed has also increased in LA, which has risen from 21.92% (control) to 31.78% (HS-30%) after 84 days (12 weeks) of the feeding period (Table 3). Several previous studies have found the same trend in the LA rate [32]. However, in other research, the LA rate did not change despite the increase in HS [15, 33]. Our results can be elaborated through the fact that the HS used are rich in ALA but also in LA (Table 1). Nevertheless, the observed increase in n-3 would possibly be responsible for a decrease in egg weight from hens (data not shown). Gonzalez-Esquerra and Leeson [34] as well as Castillo-Badillo et al. [35] observed a reduction in egg weight in treatments including tuna oil or menhaden oil in hen feed layers. This decline would be a result of low blood triglyceride levels, which restricts the amount of lipids available for the development of the egg yolk, as well as from modified estradiol circulation, which would also have an impact on the development of the egg [36]. However, there is currently no clear explanation of how n-3 FAs decrease egg weight. We also note an increase in the rate of another n-6 (GLA), whose level ranges from 0.15% (control) to 0.35% (HS-30%) (Table 3). This increase, which has been shown also in previous studies [29], is explained by the presence of GLA at quite high levels in the cannabis seeds incorporated into food. Indeed, one of the most important characteristics of cannabis seeds is their relatively high GLA content, compared to other oilseeds traditionally used in laying hen feeds, such as soybean or sunflower. Even though LA is the precursor for the synthesis of the long-chain n-6 fatty acids gamma-linolenic acid (GLA), dihomogamma-linolenic acid (DGLA), and arachidonic acid (ARA), none of these n-6 acids react in the same way. LA and ARA promote inflammation, while GLA, through its conversion to DGLA, can reduce inflammation [37]. ARA, which is derived from the elongation and desaturation of GLA, does not exhibit any change with a rate of around 2%, regardless of the diet of the animals, as we have already indicated. The same result had been obtained by other authors [38, 39], who supported their findings by pointing out that the significant increase in ALA levels promotes the n-3 pathway at the detriment of the n-6 pathway; both ways use the same enzymes, in particular the *Δ*5 and *Δ*6 desaturases. The balance of the metabolic pathway depends on the most bioavailable precursor. An excess of n-6 will promote the synthesis of ARA and DPA (C22:5n-6) to the detriment of EPA and DHA. On the other hand, a sufficient amount of n-3 will promote the synthesis of EPA and DHA and inhibit the synthesis of long-chain n-6. Moreover, at the end of the experimentation (day 84), the results show a very significant (*p* < 0.05) decrease in the n-6/n-3 ratio which goes from 8.19 (control) to 9.54, 6.15, and 4.89 for HS-10%, HS-20%, and HS-30%, respectively (Table 4). All previous studies have found the same phenomenon with usual marked decreases. For example, Mierliță [30] obtained a reduction of 11.07% (control) to only 2.98%, while Shahid et al. [39] obtained a decrease of 16.83% to 4.22%. These differences may be explained by differences in the experimental protocol and duration, or they might be linked to the variety or subspecies of the *Cannabis sativa* used. In almost all previous experiments, the seed cannabis used belongs to the industrial hemp variety; however, in our experiment, we used the *Beldiya* ecotype, which is native to the north of Morocco and is also part of the plants cataloged as nationally prohibited drugs since its THC content exceeds 0.4%.

Results, resumed in Tables 3 and 5, show that regardless of the proportion of cannabis seeds supplied to the poultry diet, the rate of SFA does not significantly change. The main SFA is the palmitic acid (C16:0), its rate slightly decreases (*p* < 0.05) from 24.38% (control) to 22.46% (HS-30%), and this reduction is partially offset by an increase in the stearic acid (C18:0) rate, which rises from 8.98% (control) to 11.17% (HS-30%). The rate of MUFA decreases significantly (*p* < 0.05) passing from 38.51 (control) to 24.42% (HS-30%). This decrease in MUFA specifically affects oleic acid (C18:1). Considering that, in fatty acid metabolism, C16:0 gives way to C18:0 via elongation and eventually to C18:1 by desaturation, the observed fluctuations are now well known. An interesting meta-analysis conducted by Fabro et al. [29], which encapsulates the majority of scientific studies investigating the effects of incorporating seeds, oil cakes, or cannabis oil on the fatty acid profile of egg yolk, allows us to validate our findings. In fact, throughout the majority of the publications reviewed in this meta-analysis, the SFA rate does not change while the MUFA rate decreases. These variations are explained by the fact that a high level of n-3 (provided by cannabis seeds) might inhibit the expression of the gene coding for stearoyl-CoA desaturase (*Δ*9 desaturases) which reduces the conversion of C18:0 to C18:1, leading to a decrease in C18:1 and an increase in C18:0.

The addition of Beldiya cannabis seed HS did not lead to significant differences in cholesterol content.  Table 7 shows that this content ranges between 7.99 and 10.73 mg/g of egg yolk, which is slightly lower than the values reported by Mierliță [30], who did not observe any differences after the cannabis-incorporated diet. However, other studies, including those conducted by Skřivan et al. [32] and Shahid et al. [40] had obtained a reduction in cholesterol content that decreased from 19.25 (control) to 11.67 mg/g of egg yolk (HS-25%). According to these authors, this decrease is due to cannabis seeds containing a high concentration of phytosterol, specifically sitosterol, which limits cholesterol absorption through crystallization and coprecipitation, but most importantly through competition at the intestinal absorption level. Kovacs et al. [41] revealed that the cholesterol content of eggs varied according to the species, variety, or stress of the laying cycle rather than the diet.

### 3.2. Tocopherol Composition

The HPLC-DAD analysis of tocopherols from lipid egg yolk (Table 8) revealed that the main tocopherols detected correspond to the isomers *α*- and *γ*-isomers, while the *β*- and *δ*-isomers were not detected. These results are consistent with those of several other subsequent studies: Ko et al. [42], Skřivan et al. [32], and Moghadam et al. [43]. However, other authors including Cherian et al. [44] and McLaughlin and Weihrauch [45] were able to detect all 4 isomers. Regardless of the treatment (age and dose), *α*-tocopherol is the major isomer constituting between 56.03% and 96.6% of total tocopherols while the percentage of *γ*-tocopherol ranges from 3.34 to 43.97% of total tocopherols. Several publications refer to preferential intakes of the *α*-isomer compared to other isoforms in mammals [46, 47] and poultry [44]. This preference is explained by the presence of a specific protein that binds *α*-tocopherol in hepatocytes and its subsequent transfer by LDL to other tissues, as well as by the potential existence of selection processes that discriminate the uptake or accumulation of *γ*-tocopherol over *α*-tocopherol. Results (Tables 8 and 9) show that the total tocopherol content varies depending on the age and dose of cannabis seeds incorporated into the diet (Table 9). According to Wang et al. [48], the main factors influencing the tocopherol content of egg yolk are nutrition, age, and laying hen variety or genotype. A significant increase in total tocopherol content depending on the dose of cannabis seeds incorporated is observed only after 84 days of treatment varying from 281.45 (control) to 327.03 *μ*g/g egg yolk (HS-30%) (Tables 8 and 9). Thus, 28 days of treatment appear, according to our results, insufficient for the fortification of eggs produced with tocopherols. An in-depth analysis of the composition of the two detected isomers (alpha and gamma) reveals dissimilar variations. So, the eggs produced are enriched with alpha-tocopherol during the treatment both in the control and for the different doses of cannabis seeds added varying from 50.52 (control) and 36.3 (HS-30%) after 28 days to 272.03 (control) and 306.3 *μ*g/g egg yolk (HS-30%) after 84 days. However, the most important outcome, which can be derived from the data obtained, is in particular the stabilization of the *γ*-tocopherol content. Indeed, regardless of the duration of treatment, the amount of *γ*-tocopherols is of the order 16.54-32.9 *μ*g/g yolk in eggs produced after the inclusion of various doses of cannabis seeds, whereas in the control eggs, there is a very significant decrease in the *γ*-tocopherol content since there is an apparent drop from 25.01 (28 days) to only 9.41 *μ*g/g yolk after 84 days of treatment. Few studies have investigated the impact of age on the incorporation of tocopherols in egg yolk. Furthermore, Tang et al. [49] observed a significant age-related increase in *α*-tocopherol from 92.984 *μ*g/g egg yolk (24 weeks) to 225.0784 *μ*g/g egg yolk (36 weeks), as well as constant values of *γ*-tocopherols (11 to 9.84 *μ*g/g egg yolk). On the other hand, Ko et al. [42] found that the level of *α*- and *γ*-tocopherols decreased with age in laying hens. According to Chen et al. [50], the concentration of tocopherols increases with age until the 14th day of the experiment, where it becomes stable. It is not entirely clear from the articles published the reason behind the differences in the tocopherol content in accordance to age. It may be due to variations in the extraction and measurement of tocopherol methods or even due to a metabolic state that is more active or less active depending on age and experimental conditions. It can also be noted that after a 28- or 84-day experiment, the increase in the amount of cannabis seeds in the diet does not lead to an increase in *γ*-tocopherol level (Table 8). Quite the contrary, a slight, nonsignificant decrease can be observed; it could be explained by poor absorption of *γ*-tocopherol from cannabis seeds, which at high concentrations contains a high amount of fiber and other antinutritional compounds which decreases and hinders the absorption of various nutrients including tocopherols.

Since animals are unable to synthesize tocopherols, provided exclusively by the diet [51], and since the vitamin premixes used did not contain vitamin E, the tocopherols came only from the dietary components of the different formulations. The increase and stability in *γ*-tocopherol content, compared to the control group, can therefore be explained by an enrichment of the diet with *γ*-tocopherols since the Beldiya cannabis seeds used contain approximately 21 times *γ* more than *α*-tocopherol [21]. Chen et al. [50] already showed that *α*-tocopherol has a positive effect on the stability of the yolk as an antioxidant at different degrees of concentration, 25, 45, and 50 *μ*g/g of yolk, yet, it turns into a prooxidant at 75 *μ*g/g and higher. Skřivan et al. [32] conducted a similar 12-week study with different hemp seed varieties, whereas in our study, we used indigenous North Moroccan *Cannabis sativa* L. seeds cultivated for their recreational use. Moreover, Skřivan et al. [32] have also shown a significant increase in the *α*-tocopherol content of egg yolk products when 60 g of cannabis seeds/kg was incorporated into the diet. On the other hand, the level of *γ*-tocopherol increased gradually in the control group, rising from 11 mg/kg MS to 29, 39, and 43 mg/kg MS for cannabis seed incorporation rates of 30, 60, and 90 g/kg, respectively. Similarly, studies conducted on diets supplemented with vitamin E [52, 53] or *α*-tocopherol [54] have shown an increase in the tocopherol content of egg yolks.

### 3.3. Chemometric Analysis of the Yolk Egg Composition

**Figure 1:**
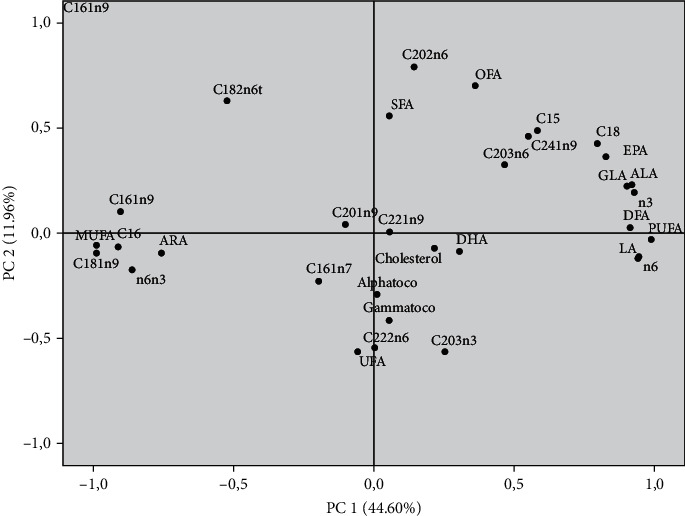
Projection of fatty acid composition, cholesterol, and tocopherol in the plane defined by two principal components. SFA: saturated fatty acids; UFA: unsaturated fatty acids; PUFA: polyunsaturated fatty acids; DFA: desirable fatty acids (C18:0+UFA); OFA: odd fatty acids; n-6: n-6 PUFA; n-3: n-3 PUFA; Alphatoco: alpha-tocopherol; Gammatoco: gamma tocopherol.

A principal component analysis (PCA) was performed to identify and determine the correlations between the hens and their yolk egg composition. The PCA allows further exploration of the above results. This analysis was conducted using 32 variables, including fatty acid profile, cholesterol content, and tocopherol composition. It provides an intelligible visualization of the relationship between hens and their egg's fatty acid profile, cholesterol, and tocopherol content.  Figure 1 presents the projection of the different elements in the plane defined by the two first principal components (PC).

**Figure 2:**
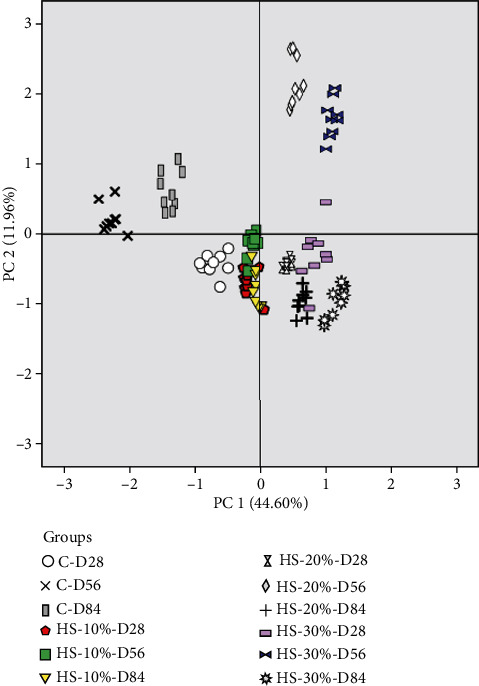
Projection of the variables of the four studied groups in the plane defined by two principal components. C-D28: control day 28; C-D56: control day 56; C-D84: control day 84; HS-10%-D28: hemp seed 10% day 28; HS-10%-D56: hemp seed 10% day 56; HS-10%-D84: hemp seed 10% day 84; HS-20%-D28: hemp seed 20% day 28; HS-20%-D56: hemp seed 20% day 56; HS-20%-D84: hemp seed 20% day 84; HS-30%-D28: hemp seed 30% day 28; HS-30%-D56: hemp seed 30% day 56; HS-30%-D84: hemp seed 30% day 84.

The PC1 and PC2 accounted for 44.60% and 11.96% of the variation, accordingly (Table 10). The PC1 was correlated positively with PUFA, PUFA n-3, PUFA n-6, ALA, and LA and negatively with MUFA and C16:0. The PC2 was characterized positively by C20:2n6, *trans*-C18:2n6, and SFA. In opposite direction, it was defined by C20:3n3 and UFA. The projection of the studied groups on the plane defined by the two first PC shows clear discrimination between hens based on egg composition (Figure 2). Regardless of the period, the hens of the control groups were located on the left side of Figures 1 and 2 differentiated from the others. However, the SH-20% and SH-30% groups were on the right side of Figure 1, with PUFA, PUFA n-3, PUFA n-6, ALA and LA. The discrimination between the animals under study shows a significant difference which is certainly linked to the impact of hemp seed incorporation in the diet of laying hens.

## 4. Conclusion

Based on the results of these experiments, we can conclude that the cannabis seed ecotype *Beldiya*, grown in the Moroccan Rif mountains, can be promoted and valued by using it as a supplement in the diet of poultry to replace, at least partially the oilseeds (soya and sunflower) imported by Morocco at a high expense. This application of cannabis in the hen's diet would provide Moroccan consumers with higher nutritional quality eggs enriched with ALA and tocopherols. Despite being advantageous nutritionally, enriching eggs with PUFAs can unfortunately promote lipid changes (increase unsaturation), leading to undesirable or even toxic products. For this reason, 20% of HS incorporation in laying hen feed is considered as a safe dose. Further studies are required to see whether adding natural antioxidants would be feasible in order to prevent these flaws in lipid oxidation and enhance the oxidative stability of the produced eggs.

## Figures and Tables

**Table 1:** Chemical composition of Beldiya ecotype hemp seed used in this study.

Elements	Percentage of fresh matter
Dry matter	88.27
Total phosphorus^∗^	11.60
Total proteins	22.00
Lipids	33.00
Calcium	1.725
Humidity	11.73
Minerals	49.70
Crude cellulose	14.68
Fatty acids (g/100 g fatty acids)	
C16:0	7.680
C18:1n9	18.050
C18:2n6	51.010
C18:3n3	16.460
Saturated fatty acids	11.910
Unsaturated fatty acids	88.090
Monounsaturated fatty acids	19.720
Polyunsaturated fatty acids	68.380

^∗^Total phosphorus on mineral element content of hemp seed.

**Table 2:** Dry matter composition (%) and nutritional composition.

Diet ingredient	Control	HS-10%	HS-20%	HS-30%
Hemp seed	0	10	20	30
Sunflower meal	1.685	13.000	13.000	13.000
Soybean meal	25.585	10.459	9.563	11.094
Calcium	8.609	7.379	8.934	8.927
DDGS	0.896	7.000	7.000	3.572
Corn	58.829	46.214	38.001	21.294
Dicalcium phosphate	1.368	3.726	1.162	2.000
Soybean oil	2.028	1.000	0.000	0.000
Premix 1	0.500	0.500	0.611	8.000
Sodium sulfate	0.000	0.237	0.300	0.200
Salt	0.183	0.112	0.200	0.200
DL-methionine	0.273	0.215	0.217	0.200
L-Lysine HCl	0.004	0.146	0.999	1.500
Premix 2	0.040	0.012	0.013	0.013
Total	100	100	100	100
Dry matter (%)	88.312	88.536	86.682	81.242
Calculated nutritional composition				
Metabolizable energy (kcal kg^−1^)	2989.920	3000.000	3000.002	3000.003
Humidity (%)	11.195	10.972	10.352	9.537
Crude protein (%)	17.799	17.800	18.000	18.000
Total lipids (%)	5.057	7.143	8.480	11.102
Ash (g/kg)	126.960	140.863	189.627	244.972
Calcium (g/kg)	38.996	40.000	40.000	40.000
Phosphorus available (g/kg)	4.396	8.510	27.762	27.752
Sodium (g/kg)	0.083	0.160	0.160	0.160
Linoleic acid (%)	2.491	7.454	12.092	17.370
Lysine (g/kg)	9.352	8.642	8.705	8.735
Methionine (g/kg)	5.580	5.589	5.823	6.138
Leucine (g/kg)	14.921	14.528	14.140	13.522
Methionine+cysteine (g/kg)	8.779	8.638	8.576	8.616
Threonine (g/kg)	6.902	6.634	6.944	6.913
Tryptophan (g/kg)	1.760	1.826	1.944	1.917

DDGS: distiller's dried grains with soluble; premix 1: vitamin premix; premix 2: mineral premix.

**Table 3:** Effect of Moroccan nonindustrial hemp seed incorporation in the diet of laying hens on fatty acid profile of egg yolk during the experimental period.

	Day 28				Day 56				Day 84			
	Control	HS-10%	HS-20%	HS-30%	Control	HS-10%	HS-20%	HS-30%	Control	HS-10%	HS-20%	HS-30%
C8:0	0.002^ab^ ± 0.001	0.003^b^ ± 0.002	0.0032^b^ ± 0.006	0.003^ab^ ± 0.001	0.002^ab^ ± 0.000	0.003^b^ ± 0.002	0.002^ab^ ± 0.001	0.002^ab^ ± 0.000	0.002^ab^ ± 0.000	0.001^a^ ± 0.000	0.001^a^ ± 0.000	0.001^a^ ± 0.001
C11:0	0.003^a^ ± 0.002	0.005^a^ ± 0.002	0.005^a^ ± 0.000	0.006^a^ ± 0.004	0.015^b^ ± 0.002	0.004^a^ ± 0.001	0.006^a^ ± 0.002	0.004^a^ ± 0.002	0.020^c^ ± 0.006	0.018^a^ ± 0.001	0.014^b^ ± 0.003	0.011^c^ ± 0.006
C12:0	0.004^abc^ ± 0.000	0.004^abc^ ± 0.00	0.003^a^ ± 0.000	0.004^d^ ± 0.001	0.004^cd^ ± 0.000	0.003^ab^ ± 0.000	0.005^e^ ± 0.000	0.006^f^ ± 0.000	0.004^cd^ ± 0.000	0.003^a^ ± 0.00	0.003^a^ ± 0.001	0.003^a^ ± 0.000
C14:0	0.242^abc^ ± 0.018	0.255^abcde^ ± 0.015	0.269^e^ ± 0.007	0.258^cde^ ± 0.005	0.268^de^ ± 0.005	0.239^ab^ ± 0.012	0.249^abc^ ± 0.006	0.236^a^ ± 0.007	0.266^de^ ± 0.020	0.256^bcde^ ± 0.019	0.266^de^ ± 0.007	0.249^abc^ ± 0.008
C14:1	0.335^b^ ± 0.006	0.035^b^ ± 0.001	0.022^a^ ± 0.002	0.036^b^ ± 0.012	0.547^d^ ± 0.003	0.020^a^ ± 0.004	0.074^e^ ± 0.008	0.046^c^ ± 0.006	0.535^cd^ ± 0.016	0.033^b^ ± 0.002	0.033^b^ ± 0.006	0.022^a^ ± 0.003
C15:0	0.089^bcd^ ± 0.005	0.077^ab^ ± 0.001	0.088^bc^ ± 0.004	0.103^d^ ± 0.022	0.075^ab^ ± 0.005	0.069^a^ ± 0.005	0.084^bc^ ± 0.005	0.128^e^ ± 0.015	0.066^a^ ± 0.003	0.075^ab^ ± 0.006	0.083^bc^ ± 0.012	0.092^cd^ ± 0.005
C15:1	0.006^d^ ± 0.002	0.003^abc^ ± 0.002	0.003^bc^ ± 0.002	0.002^abc^ ± 0.002	0.002^abc^ ± 0.001	0.002^abc^ ± 0.001	0.001^ab^ ± 0.000	0.000^a^ ± 0.000	0.004^cd^ ± 0.000	0.003^abc^ ± 0.002	0.002^abc^ ± 0.002	0.003^abc^ ± 0.002
C16:0	23.258^ef^ ± 0.523	23.639^f^ ± 0.426	23.153^de^ ± 0.072	22.728^bcd^ ± 0.167	25.463^i^ ± 0.313	23.384^efg^ ± 0.196	22.680^abc^ ± 0.153	22.209^a^ ± 0.153	24.381^h^ ± 0.430	23.808^g^ ± 0.168	23.106^cde^ ± 0.178	22.461^ab^ ± 0.301
C16:1n9	1.824^c^ ± 0.123	1.037^b^ ± 0.073	1.047^b^ ± 0.056	0.900^ab^ ± 0.025	2.815^e^ ± 0158	0.997^ab^ ± 0.057	1.015^ab^ ± 0.070	0.883^a^ ± 0.077	2.382^d^ ± 0.173	0.998^ab^ ± 0.077	1.033^b^ ± 0.082	0.911^ab^ ± 0.054
C16:1n7	0.743^b^ ± 0.031	0.585^ab^ ± 0.254	0.590^ab^ ± 0.190	0.620^b^ ± 0.167	0.677^b^ ± 0.040	0.731^b^ ± 0.160	0.358^a^ ± 0.141	0.561^ab^ ± 0.202	0.616^ab^ ± 0.019	0.548^ab^ ± 0.227	0.573^ab^ ± 0.183	0.586^ab^ ± 0.164
C17:0	0.008^bc^ ± 0.001	0.009^de^ ± 0.001	0.009^e^ ± 0.001	0.009^de^ ± 0.001	0.008^cde^ ± 0.001	0.008^cd^ ± 0.001	0.006^a^ ± 0.001	0.009^de^ ± 0.001	0.006^ab^ ± 0.001	0.006^ab^ ± 0.000	0.007^ab^ ± 0.001	0.007^abc^ ± 0.001
C17:1	0.003^abc^ ± 0.001	0.005^cd^ ± 0.001	0.003^abc^ ± 0.001	0.003^abc^ ± 0.001	0.002^ab^ ± 0.000	0.006^de^ ± 0.001	0.002^a^ ± 0.000	0.002^ab^ ± 0.000	0.004^cd^ ± 0.001	0.007^e^ ± 0.001	0.004^bcd^ ± 0.004	0.012^f^ ± 0.000
C18:0	9.603^b^ ± 0.172	9.744^bc^ ± 0.084	10.713^e^ ± 0.224	12.689^h^ ± 0.225	9.112^a^ ± 0.163	9.797^bc^0.312	10.319^d^ ± 0.181	11.922^g^ ± 0.215	8.980^a^ ± 0.117	9.813^bc^ ± 0.089	9.991^c^ ± 0.082	11.170^f^ ± 0.127
C18:1n9	30.853^e^ ± 0.315	28.433^d^ ± 0.373	25.197^b^ ± 0.271	21.844^a^ ± 0.224	37.187^g^ ± 0.762	27.925^cd^ ± 0.240	24.824^b^ ± 0.266	21.802^a^ ± 0.151	33.984^f^ ± 0.662	27.421^c^ ± 0.526	24.695^b^ ± 0.243	21.850^a^ ± 0.202
C18:1n7	0.911^a^ ± 0.051	0.844^a^ ± 0.362	0.807^a^ ± 0.136	0.821^a^ ± 0.232	1.143^b^ ± 0.018	0.840^a^ ± 0.135	1.434^b^ ± 0.224	1.039^a^ ± 0.145	1.147^b^ ± 0.174	1.027^a^ ± 0.282	1.065^a^ ± 0.190	1.043^a^ ± 0.166
C18:2n6t	0.024^b^ ± 0.002	0.025^bc^ ± 0.001	0.022^b^ ± 0.002	0.026^bc^ ± 0.008	0.043^d^ ± 0.002	0.022^b^ ± 0.003	0.047^d^ ± 0.005	0.032^c^ ± 0.008	0.040^d^ ± 0.005	0.024^b^ ± 0.004	0.019^b^ ± 0.003	0.012^a^ ± 0.003
C18:2n6 (LA)	25.454^c^ ± 0.464	29.108^d^ ± 0.242	29.999^e^ ± 0.173	30.554^f^ ± 0.253	17.922^a^ ± 0.599	29.859^e^ ± 0.389	30.709^f^ ± 0.234	31.454^gh^ ± 0.233	21.923^b^ ± 0.440	29.911^e^ ± 0.226	31.020^fg^ ± 0.237	31.785^h^ ± 0.166
C18:3n6 (GLA)	0.181^c^ ± 0.020	0.219^c^ ± 0.007	0.288^d^ ± 0.019	0.353^e^ ± 0.025	0.160^ab^ ± 0.003	0.225^c^ ± 0.021	0.288^c^ ± 0.021	0.350^e^ ± 0.029	0.145^a^ ± 0.005	0.232^c^ ± 0.023	0.292^d^ ± 0.017	0.348^e^ ± 0.033
C18:3n3 (ALA)	1.898^c^ ± 0.070	1.932^c^ ± 0.051	3.693^d^ ± 0.179	5.014^f^ ± 0.271	0.549^a^ ± 0.029	1.968^c^ ± 0.091	3.815^de^ ± 0.128	5.248^g^ ± 0.049	1.623^b^ ± 0.162	1.966^c^ ± 0.061	3.938^e^ ± 0.066	5.468^h^ ± 0.052
C20:0	0.025^cd^ ± 0.002	0.030^de^ ± 0.004	0.030^de^ ± 0.002	0.027^cd^ ± 0.008	0.036^e^ ± 0.011	0.027^cd^ ± 0.003	0.021^bc^ ± 0.003	0.021^c^ ± 0.003	0.021^bc^ ± 0.005	0.0134^ab^ ± 0.001	0.029^cde^ ± 0.007	0.010^a^ ± 0.001
C20:1n9	0.012^e^ ± 0.001	0.015^f^ ± 0.002	0.005^ab^ ± 0.001	0.010^de^0.003	0.008^bcd^0.001	0.005^a^ ± 0.001	0.008^cd^ ± 0.001	0.006^abc^ ± 0.000	0.006^abc^ ± 0.000	0.005^ab^ ± 0.003	0.007^abc^ ± 0.002	0.006^abc^ ± 0.003
C20:2n6	0.019^abc^ ± 0.002	0.024^bcd^ ± 0.005	0.030^cde^ ± 0.002	0.034^de^ ± 0.004	0.044^e^ ± 0.004	0.024^bcd^ ± 0.002	0.126^f^ ± 0.023	0.101^g^ ± 0.018	0.025^bcd^ ± 0.002	0.015^ab^ ± 0.001	0.012^ab^ ± 0.001	0.009^a^ ± 0.001
C21:0	0.049^bc^ ± 0.005	0.052^bc^ ± 0.006	0.052^bc^ ± 0.005	0.050^bc^ ± 0.006	0.057^c^ ± 0.003	0.056^c^ ± 0.009	0.119^f^ ± 0.012	0.074^e^ ± 0.011	0.058^c^ ± 0.008	0.051^bc^ ± 0.001	0.042^ab^ ± 0.006	0.033^a^ ± 0.004
C20:3n6	0.215^ab^ ± 0.017	0.220^ab^ ± 0.045	0.292^c^ ± 0.019	0.239^ab^ ± 0.050	0.207^a^ ± 0.019	0.242^ab^ ± 0.033	0.259^bc^ ± 0.027	0.290^c^ ± 0.021	0.215^ab^ ± 0.040	0.224^ab^ ± 0.040	0.208^a^ ± 0.030	0.258^bc^ ± 0.021
C20:4n6 (ARA)	2.695^d^ ± 0.115	2.144^bc^ ± 0.094	2.028^ab^ ± 0.069	1.988^a^ ± 0.035	2.623^d^ ± 0.087	2.070^ab^ ± 0.153	1.970^a^ ± 0.063	1.960^a^ ± 0.035	2.283^c^ ± 0.091	2.048^ab^ ± 0.059	1.980^a^ ± 0.114	1.992^a^ ± 0.100
C20:3n3	0.006^a^ ± 0.001	0.014^b^ ± 0.002	0.0125^ab^ ± 0.002	0.013^ab^ ± 0.005	0.018^b^ ± 0.008	0.012^ab^ ± 0.001	0.014^b^0.003	0.017^b^ ± 0.005	0.057^c^ ± 0.003	0.087^d^ ± 0.004	0.093^d^ ± 0.004	0.126^e^ ± 0.006
C22:0	0.010^b^ ± 0.001	0.013^cd^ ± 0.001	0.015^de^ ± 0.002	0.012^c^ ± 0.002	0.007^a^ ± 0.001	0.012^c^ ± 0.002	0.017^e^ ± 0.001	0.013^cd^ ± 0.003	0.008^ab^ ± 0.001	0.011^c^ ± 0.001	0.008^a^ ± 0.002	0.008^ab^ ± 0.001
C22:1n9	0.004^abc^ ± 0.000	0.004^abc^ ± 0.001	0.008^d^ ± 0.002	0.004^abc^ ± 0.001	0.004^abc^ ± 0.001	0.006^cd^ ± 0.002	0.004^abc^ ± 0.001	0.003^ab^ ± 0.001	0.003^ab^ ± 0.001	0.003^ab^ ± 0.001	0.005^bc^ ± 0.003	0.002^a^ ± 0.000
20:5n3 (EPA)	0.016^a^ ± 0.004	0.036^b^ ± 0.005	0.067^c^ ± 0.004	0.096^e^ ± 0.005	0.028^b^ ± 0.011	0.035^b^ ± 0.004	0.063^c^ ± 0.003	0.089^de^ ± 0.006	0.017^a^ ± 0.001	0.035^b^ ± 0.006	0.061^c^ ± 0.002	0.085^d^ ± 0.004
C22:2n6	0.021^c^ ± 0.004	0.023^c^ ± 0.003	0.022^c^ ± 0.002	0.015^b^ ± 0.007	0.022^c^ ± 0.002	0.003^a^ ± 0.001	0.003^a^ ± 0.001	0.002^a^ ± 0.000	0.003^a^ ± 0.000	0.023^c^ ± 0.002	0.025^c^ ± 0.006	0.024^c^ ± 0.003
C24:0	0.021^abc^ ± 0.000	0.024^abc^ ± 0.003	0.028^bc^ ± 0.005	0.025^abc^ ± 0.005	0.024^abc^ ± 0.002	0.020^abc^ ± 0.001	0.047^e^ ± 0.009	0.045^cd^ ± 0.009	0.021^abc^ ± 0.004	0.014^a^ ± 0.001	0.016^ab^ ± 0.003	0.031^cd^0.024
C24:1n9	ND	0.003^b^ ± 0.000	0.005^c^ ± 0.001	0.005^c^ ± 0.001	ND	0.003^b^ ± 0.000	0.004^bc^ ± 0.001	0.005^c^ ± 0.002	ND	ND	0.003^b^ ± 0.001	ND
22:6n3 (DHA)	1.764^c^ ± 0.238	1.435^b^ ± 0.135	1.489^b^ ± 0.103	1.506^b^ ± 0.221	1.130^a^ ± 0.032	1.383^b^ ± 0.175	1.424^b^ ± 0.156	1.439^b^ ± 0.107	1.313^ab^ ± 0.048	1.321^ab^ ± 0.118	1.364^b^ ± 0.175	1.370^b^ ± 0.040

Day 28: Week 28; day 56: week 32; day 84: week 36; HS-10%: hemp seed 10%; HS-20%: hemp seed 20%; HS-30%: hemp seed 30%; %: mean ± standard deviation. Significant differences (*p* < 0.05) between means are shown with different lowercase letters (a–h).

**Table 4:** Analysis of variance for the effect of Moroccan nonindustrial hemp seed incorporation in the diet of laying hens on fatty acid profile during the experimental period.

	df	Mean squares	*F*-value	*p* value
C18:0				
Dose	3	36.495	1138.033	<0.001
Period	2	4.425	137.981	<0.001
Dose × period	6	0.975	30.411	<0.001
Error	96	0.032		
C18:1n9				
Dose	3	728.574	4582.418	<0.001
Period	2	17.344	109.088	<0.001
Dose × period	6	25.279	158.992	<0.001
Error	96	0.159		
C18:2n-6 (LA)				
Dose	3	525.677	4792.460	<0.001
Period	2	18.368	167.455	<0.001
Dose × period	6	39.113	356.585	<0.001
Error	96	0.110		
C18:3n6 (GLA)				
Dose	3	0.178	410.749	<0.001
Period	2	0.000	0.893	<0.5
Dose × period	6	0.001	2.311	<0.050
Error	96	0.000		
C18:3n3 (ALA)				
Dose	3	85.106	5697.618	<0.001
Period	2	1.174	78.626	<0.001
Dose × period	6	1.334	89.333	<0.001
Error	96	0.015		
C20:4n-6 (ARA)				
Dose	3	1.843	222.301	<0.001
Period	2	0.172	20.750	<0.001
Dose × period	6	0.099	11.979	<0.001
Error	96	0.008		
20:5n-3 (EPA)				
Dose	3	0.026	880.377	<0.001
Period	2	0.000	7.656	0.001
Dose × period	6	0.000	6.395	<0.001
Error	96	2.929^−005^		
22:6n-3 (DHA)				
Dose	3	0.018	0.866	<0.500
Period	2	0.506	24.281	<0.001
Dose × period	6	0.186	8.921	<0.001
Error	96	0.021		

df: degree of freedom.

**Table 5:** Effect of Moroccan nonindustrial hemp seed incorporation in the diet of laying hens on the sums and the ratio of fatty acids of egg yolk during the experimental period.

	Day 28				Day 56				Day 84			
	Control	HS-10%	HS-20%	HS-30%	Control	HS-10%	HS-20%	HS-30%	Control	HS-10%	HS-20%	HS-30%
SFA	33.315^a^ ± 0.547	33.855^abc^ ± 0.444	34.370^cd^ ± 0.253	35.914^f^ ± 0.324	35.071^e^ ± 0.384	33.622^ab^ ± 0.398	33.557^ab^ ± 0.257	34.669^de^ ± 0.335	33.834^abc^ ± 0.381	34.071^bc^ ± 0.169	33.565^ab^ ± 0.241	34.088^bc^ ± 0.348
UFA	66.685^f^ ± 0.547	66.141^def^ ± 0.445	65.627^cd^ ± 0.253	64.082^a^ ± 0.324	64.929^b^ ± 0.385	66.372^ef^ ± 0.398	66.441^ef^ ± 0.257	65.328^bc^ ± 0.335	66.166^def^ ± 0.381	65.922^de^ ± 0.169	66.431^ef^ ± 0.240	65.899^de^ ± 0.348
MUFA	34.389^e^ ± 0.277	30.959^d^ ± 0.610	27.684^b^ ± 0.254	24.243^a^ ± 0.486	42.181^g^ ± 0.791	30.529^cd^ ± 0.279	27.722^b^ ± 0.201	24.344^a^ ± 0.390	38.519^f^ ± 0.670	30.038^c^ ± 0.272	27.416^b^ ± 0.370	24.424^a^ ± 0.376
PUFA	32.296^c^ ± 0.612	35.182^d^ ± 0.253	37.943^f^ ± 0.188	39.840^h^ ± 0.563	22.748^a^ ± 0.704	35.843^de^ ± 0.404	38.719^g^ ± 0.315	40.984^i^ ± 0.331	27.647^b^ ± 0.539	35.884^e^ ± 0.218	39.015^h^ ± 0.302	41.477^i^ ± 0.225
PUFA n-6	28.610^c^ ± 0.502	31.765^d^ ± 0.235	32.682^e^ ± 0.217	33.209^de^ ± 0.223	21.022^a^ ± 0.655	32.446^e^ ± 0.487	33.402^g^ ± 0.208	34.189^h^ ± 0.253	24.636^b^ ± 0.403	32.472^e^ ± 0.261	33.559^f^ ± 0.301	34.428^h^ ± 0.176
PUFA n-3	3.685^c^ ± 0.282	3.417^c^ ± 0.102	5.262^d^ ± 0.224	6.630^e^ ± 0.374	1.726^a^ ± 0.058	3.400^c^ ± 0.225	5.316^d^ ± 0.245	6.794^ef^ ± 0.120	3.011^b^ ± 0.162	3.409^c^ ± 0.138	5.456^d^ ± 0.135	7.049^f^ ± 0.725
n-6/n-3	7.801^c^ ± 0.554	9.302^d^ ± 0.295	6.222^b^ ± 0.292	5.022^a^ ± 0.262	12.185^e^ ± 0.228	9.590^d^ ± 0.691	6.295^b^ ± 0.289	5.033^a^ ± 0.077	8.198^c^ ± 0.347	9.540^d^ ± 0.427	6.155^b^ ± 0.173	4.884^a^ ± 0.042
DFA	76.288^de^ ± 0.531	75.885^cd^ ± 0.422	76.340^def^ ± 0.075	76.772^fg^ ± 0.163	74.041^a^ ± 0.308	76.169^cde^ ± 0.204	76.760^fg^ ± 0.291	77.250^h^ ± 0.159	75.146^b^ ± 0.423	75.735^c^ ± 0.169	76.422^ef^ ± 0.176	77.070^gh^ ± 0.287
OFA	0.158^abc^ ± 0.006	0.150^a^ ± 0.006	0.161^abc^ ± 0.004	0.173^c^ ± 0.021	0.159^abc^ ± 0.006	0.144^a^ ± 0.113	0.219^d^ ± 0.014	0.218^d^ ± 0.013	0.159^abc^ ± 0.013	0.161^abc^ ± 0.009	0.151^ab^ ± 0.011	0.168^bc^ ± 0.008

Day 28: week 28; day 56: week 32; day 84: week 36; HS-10%: hemp seed 10%; HS-20%: hemp seed 20%; HS-30%: hemp seed 30%; SFA: saturated fatty acids; UFA: unsaturated fatty acids; PUFA: polyunsaturated fatty acids; DFA: desirable fatty acids (C18:0+UFA); OFA: odd fatty acids; n-6: PUFA n-6; n-3: PUFA n-3. Significant differences (*p* < 0.05) between means are shown with different lowercase letters (a–f).

**Table 6:** Analysis of variance for the effect of Moroccan nonindustrial hemp seed incorporation in the diet of laying hens on fatty acid profile sum ant ratios during the experimental period.

SFA				
Dose	3	6.719	53.592	<0.001
Period	2	2.153	17.174	<0.001
Dose × period	6	5.142	41.017	<0.001
Error	96	0.125		
UFA				
Dose	3	6.744	53.798	<0.001
Period	2	2.120	16.916	<0.001
Dose × period	6	5.134	40.956	<0.001
Error	96	0.125		
MUFA				
Dose	3	970.495	4703.078	<0.001
Period	2	31.944	154.802	<0.001
Dose × period	6	35.677	172.893	<0.001
Error	96	0.206		
PUFA				
Dose	3	900.335	5049.344	<0.001
Period	2	31.078	174.296	<0.001
Dose × period	6	61.527	345.062	<0.001
Error	96	0.178		
PUFA n-6				
Dose	3	486.461	3816.600	<0.001
Period	2	16.787	131.707	<0.001
Dose × period	6	40.015	313.948	<0.001
Error	96	0.127		
PUFA n-3				
Dose	3	91.235	2289.228	<0.001
Period	2	2.237	56.123	<0.001
Dose × period	6	2.392	60.012	<0.001
Error	96	0.040		
PUFA n-6/PUFA n-3				
Dose	3	139.298	1112.580	<0.001
Period	2	15.565	124.319	<0.001
Dose × period	6	12.553	100.264	<0.001
Error	96	0.125		
DFA				
Dose	3	17.423	197.216	<0.001
Period	2	0.746	8.444	<0.001
Dose × period	6	4.008	45.366	<0.001
Error	96	0.088		
OFA				
Dose	3	0.007	52.041	<0.001
Period	2	0.007	55.367	<0.001
Dose × period	6	0.004	31.124	<0.001
Error	96	0.000		

**Table 7:** Effect of Moroccan nonindustrial hemp seed incorporation in the diet of laying hens on cholesterol content of egg yolk during the experimental period.

	Day 28				Day 56				Day 84			
	Control	HS-10%	HS-20%	HS-30%	Control	HS-10%	HS-20%	HS-30%	Control	HS-10%	HS-20%	HS-30%
Cholesterol (mg/g)	9.27 ± 0.720	8.368 ± 0.570	9.648 ± 1.002	9.698 ± 1.449	8.284 ± 3.651	9.970 ± 0.671	7.991 ± 0.836	10.739 ± 0.844	8.284 ± 3.651	9.970 ± 0.671	7.991 ± 0.836	10.739 ± 0.844

Day 28: week 28; day 56: week 32; day 84: week 36; HS-10%: hemp seed 10%; HS-20%: hemp seed 20%; HS-30%: hemp seed 30%; %: mean ± standard deviation.

**Table 8:** Effect of Moroccan nonindustrial hemp seed incorporation in the diet of laying hens on tocopherol composition of egg yolk after 28 and 84 days of the experiment.

	Day 28				Day 84			
	Control	HS-10%	HS-20%	HS-30%	Control	HS-10%	HS-20%	HS-30%
*α*-Tocopherol (*μ*g/g)	50.520^ab^ ± 3.295	40.686^ab^ ± 0.905	52.863^b^ ± 3.709	36.306^a^ ± 1.964	272.037^e^ ± 6.842	128.816^d^ ± 2.288	86.829^c^ ± 1.054	306.303^f^ ± 12.412
*γ*-Tocopherol (*μ*g/g)	25.012^bc^ ± 1.409	32.919^c^ ± 11.286	16.546^ab^ ± 0.156	21.546^abc^ ± 0.118	9.411^a^ ± 1.106	32.332^c^ ± 2.379	30.579^c^ ± 5.224	20.726^abc^ ± 0.765
*α*-Toco/*γ*-Toco	2.019^a^ ± 2.019	1.322^a^ ± 0.389	3.194^a^ ± 0.193	1.685^a^ ± 0.083	29.109^c^ ± 2.580	3.997^a^ ± 0.200	2.888^a^ ± 0.428	14.780^b^ ± 0.299
Total tocopherol (*μ*g/g)	75.535^a^ ± 4.677	73.606^a^ ± 10.183	69.408^a^ ± 3.864	57.853^a^ ± 2.079	281.447^d^ ± 7.946	161.148^c^ ± 4.228	117.409^b^ ± 5.991	327.028^e^ ± 13.085

Day 28: week 28; day 84: week 36; HS-10%: hemp seed 10%; HS-20%: hemp seed 20%; HS-30%: hemp seed 30%; *α*-Toco: *α*-tocopherol; *γ*-Toco: *γ*-tocopherol; %: mean ± standard deviation. Significant differences (*p* < 0.05) between means are shown with different lowercase letters (a–h).

**Table 9:** Analysis of variance for the effect of Moroccan nonindustrial hemp seed incorporation in the diet of laying hens on cholesterol content and tocopherol composition during the experimental period.

	df	Mean squares	*F*-value	*p* value
*α*-Toco				
Dose	3	16071.283	543.464	<0.001
Period	1	140625.354	4755.364	<0.001
Dose × period	3	18460.871	624.270	<0.001
Error	16	29.572		
*γ*-Toco				
Dose	3	275.048	275.048	<0.001
Period	1	5.927	0.289	>0.500
Dose × period	3	219.788	10.711	<0.001
Error	16	20.520		
*α*-Toco/*γ*-Toco				
Dose	3	217.636	241.916	<0.001
Period	1	679.060	754.816	<0.001
Dose × period	3	229.937	255.589	<0.001
Error	16	.900		
Total Toco				
Dose	3	13592.350	251.142	<0.001
Period	1	139826.796	2583.541	<0.001
Dose × period	3	15803.113	291.990	<0.001
Error	16	54.122		
Cholesterol				
Dose	3	6.733	2.354	>0.050
Period	2	1.370^−007^	0.000	1.000
Dose × period	6	2.456	0.859	>0.500
Error	24	2.861		

*α*-Toco: *α*-tocopherol; *γ*-Toco: *γ*-tocopherol; df: degree of freedom.

**Table 10:** Three main components explain more than 64.25% of the total information on fatty acid profile, cholesterol content, and tocopherol composition during the experimental period.

Variables	Principal component		
	1	2	3
C18:1n9	-0.986	0.101	-0.044
MUFA	-0.979	0.132	-0.028
PUFA	0.964	-0.212	-0.087
PUFA n-3	0.949	0.017	0.093
C18:3n-3 (ALA)	0.945	0.046	0.136
C18:3n-6 (GLA)	0.930	0.046	0.188
C16:00	-0.907	0.120	0.301
PUFA n-6	0.904	-0.291	-0.155
DFA	0.902	-0.151	-0.296
C18:2n6 (LA)	0.901	-0.296	-0.149
EPA	0.885	0.202	0.310
n-6/n-3	-0.879	-0.003	0.032
C16:1n9	-0.865	0.280	0.108
C18:0	0.863	0.267	0.331
C20:4n-6 (ARA)	-0.761	0.059	-0.020
C15:0	0.667	0.370	0.118
C24:1n9	0.628	0.352	-0.079
C20:3n6	0.521	0.230	-0.050
C20:2n6	0.291	0.753	-0.440
C18:2n6t	-0.390	0.723	-0.278
OFA	0.492	0.622	-0.281
C22:2n6	-0.106	-0.535	0.438
*γ*-Tocopherol	-0.025	-0.418	0.174
UFA	-0.166	-0.538	-0.791
SFA	0.164	0.541	0.789
C20:3n-3	0.139	-0.602	0.301
*α*-Tocopherol	-0.044	-0.288	0.174
C20:1n9	-0.089	0.059	-0.125
C22:1n9	0.057	-0.005	-0.060
DHA	0.280	-0.140	-0.409
C16:1n7	-0.237	-0.183	0.118
Cholesterol	0.198	-0.112	0.137

SFA: saturated fatty acids; UFA: unsaturated fatty acids; PUFA: polyunsaturated fatty acids; DFA: desirable fatty acids (C18:0+UFA); OFA: odd fatty acids; n-6: PUFA n-6; n-3: PUFA n-3.

## Data Availability

The original data from the paper are available from the corresponding author upon reasonable request.
